# IN-VEHICLE SENSORS TO DETECT CHANGES IN DRIVING BEHAVIORS WITH EMERGENCE OF MCI

**DOI:** 10.1093/geroni/igad104.2099

**Published:** 2023-12-21

**Authors:** Ruth Tappen, Jinwoo Jang, Sonia Moshfeghi, KwangSoo Yang, Monica Rosselli, Joshua Coniff, Borko Furht, David Newman

**Affiliations:** Florida Atlantic University, Boca Raton, Florida, United States; Florida Atlantic University, Boca Raton, Florida, United States; Florida Atlantic University (FAU), Boca Raton, Florida, United States; Florida Atlantic University (FAU), Boca Raton, Florida, United States; Florida Atlantic University (FAU), Boca Raton, Florida, United States; Florida Atlantic University (FAU), Boca Raton, Florida, United States; Florida Atlantic University (FAU), Boca Raton, Florida, United States; Florida Atlantic University, Boca Raton, Florida, United States

## Abstract

Driving is a complex behavior requiring attention, judgment, orientation, memory, processing speed, visual acuity, adequate limb strength, and other functional abilities. It has been suggested that a change in driving behavior may be an early indicator of cognitive decline in the aging brain. The literature suggests that older drivers will drive less and more slowly as their cognitive skills begin to decline. In the “In-Vehicle Sensors to Detect Cognitive Change in Older Drivers” study, sensors are installed in participant vehicles and data is downloaded every three months at which time a carefully curated battery of cognitive tests is also administered. Records of those suggesting mild cognitive impairment (MCI) are reviewed by a Consensus Diagnostic Panel for confirmation. In the first 100 participants who have been followed over a year, nine participants evidenced existing or emerging MCI. Of these, six have experienced a decline in cognition in the last year. A comparison of driving behaviors over the year evidenced the previously reported slower, less frequent driving but also a previously unreported increase in hard braking and hard turns. The uniqueness of our longitudinal study is the continuous tracking of vehicle acceleration patterns over time, revealing significant relationships between g-force acceleration and cognition.

